# Creutzfeldt-Jakob Disease: A Rare Case of Dementia

**DOI:** 10.7759/cureus.47177

**Published:** 2023-10-17

**Authors:** Cláudio Gouveia, Luís M Morais, Susana Guimarães, Cristiana Camacho, Susana Jesus

**Affiliations:** 1 Internal Medicine, Centro Hospitalar Lisboa Ocidental, Lisbon, PRT; 2 Critical Care Medicine, Centro Hospitalar Lisboa Ocidental, Lisbon, PRT; 3 Pathology, Centro Hospitalar São João, Porto, PRT

**Keywords:** case report, cognitive decline, dementia, prion disease, creutzfeld-jakob disease

## Abstract

Prion diseases are rare neurodegenerative diseases that have a rapid evolution. Creutzfeldt-Jakob disease (CJD) is the most common and its sporadic form the most frequent. Definitive diagnosis is only obtained through autopsy, and there are currently no available treatments. Here, we present a case of an 84-year-old woman presenting with resting tremor, abnormal gait, frequent falls, apraxia, visual hallucinations, and delirium. There were no signs of relevant metabolic, infectious, or nutritional alterations, and brain computed tomography (CT) scan and magnetic resonance imaging (MRI) had no significant findings. Two months later, the patient was completely immobile with mutism, seizures, and myoclonus. In the presence of a rapidly progressive dementia associated with myoclonus, it was hypothesized that the patient had CJD. The patient’s clinical state deteriorated, she died, and autopsy confirmed sporadic CJD. The purpose of this case is to highlight a rare disease that can go undiagnosed because of low awareness and clinical suspicion and the importance of the differential diagnosis of dementia, a common disease at this age.

## Introduction

The increase in longevity and survival has caused a rise in the prevalence of chronic diseases in advanced ages, such as dementia [1]. Dementia, as a clinical syndrome, has many potential causes. The majority of cases are neurodegenerative and/or vascular with relatively gradual progressive clinical courses. However, dementia can be rapidly progressive, and different aetiologies must be considered, namely, infections, metabolic and immune disorders, neoplasms, and nutritional deficiencies [2].

Prion diseases are neurodegenerative diseases that have long incubation periods and progress inevitably to death once clinical symptoms appear. Creutzfeldt-Jakob disease (CJD) is the most common human prion disease [3]. Of the various forms of CJD (sporadic, iatrogenic, genetic, and variant), the sporadic form is the most frequent [4]. It is typically characterized by a rapidly progressive clinical course and widespread brain deposition of abnormal prion protein aggregates leading to spongiform change, gliosis, and neuronal loss [3].

The mean age for disease onset is between 57 and 62 years; however, cases in young adults and those over 80 years of age have also been described [5]. Clinically, it consists of rapidly progressive dementia with cerebellar ataxia, visual disturbances, and myoclonus terminating in an akinetic mute state. Clinical presentation can be varied and unspecific, making the diagnosis very difficult and challenging [6]. In older patients, a coexistence with other dementias, mainly vascular dementia and Alzheimer's disease, is not uncommon [7].

As for the diagnosis, imaging studies, electroencephalography (EEG), and biomarkers are used in conjunction with the clinical picture to try to establish it. Out of all the imaging options, magnetic resonance imaging (MRI) is the most helpful technique for diagnosing CJD. When it comes to an electroencephalogram (EEG), a characteristic pattern is periodic biphasic and triphasic sharp-wave complexes, although these have low sensitivity for diagnosis. Cerebrospinal fluid (CSF) analysis plays an important role in the diagnosis, since the detection of abnormal 14.3.3 protein levels is helpful for the differential diagnosis, representing a strong supportive diagnostic tool. Microtubule-associated protein tau is also largely used as a marker for the premortem diagnosis of CJD, having shown comparable sensitivity and specificity as protein 14.3.3. However, histopathological confirmation is considered the gold standard for definitive diagnosis [8].

There is no effective treatment available at present, so treatment is merely supportive, and CJD is always fatal. However, there are ongoing investigations on treatment options for CJD and other prion diseases [9].

## Case presentation

An 84-year-old woman, with a history of hypertension and asthma came to the emergency department (ED) presenting with a resting tremor, abnormal gait with frequent falls, apraxia, visual hallucinations, and delirium that had developed and worsened over the prior month. The patient’s family reported that there was already a history of insomnia, anterograde amnesia, and temporospatial disorientation with a progressive but fast loss of autonomy over the previous year. Physical examination revealed only disorientation and psychomotor retardation with no other relevant neurological findings. Blood analysis revealed normocytic and normochromic anemia with a hemoglobin of 10.9g/dL (12.0-15.0g/dL) and a hyponatremia of 128 mmol/L (136-145 mmol/L), with no signs of infection. Brain computed tomography (CT) scan excluded acute ischemic or hemorrhagic lesions. An acute decompensation of an underlying dementia was considered the most probable diagnosis, and the patient was admitted for treatment and further investigation. Thyroid function was also assessed and was normal, and there were no other ionic or vitamin deficiencies. HIV and syphilis serologies were negative. MRI had no relevant findings. The patient evolved favorably, and after sodium correction, she was discharged from the hospital.

Two months later, the patient was brought to the ED for a pulmonary infection. She was completely immobile, confined to bed with mutism, and had developed focal onset impaired awareness seizures and right arm myoclonus. The patient was admitted to the internal medicine ward for intravenous antibiotic treatment. In the presence of a rapid progression of the patient’s dementia, with a total loss of autonomy in less than a year, associated with myoclonus, the diagnosis of CJD was considered. The EEG showed isolated and periodic biphasic and triphasic waves with one to two second repetition. The CSF analysis revealed a slightly elevated cell count (6.0/mm^3^, with no clear cell predominance) with elevated proteins (144.4 mg/dL). Fluid cultures were negative, but the CSF was positive for protein 14.3.3, with elevated total tau protein (13200 pg/mL) and normal phosphorylated tau protein (67 pg/mL).

**Figure 1:**
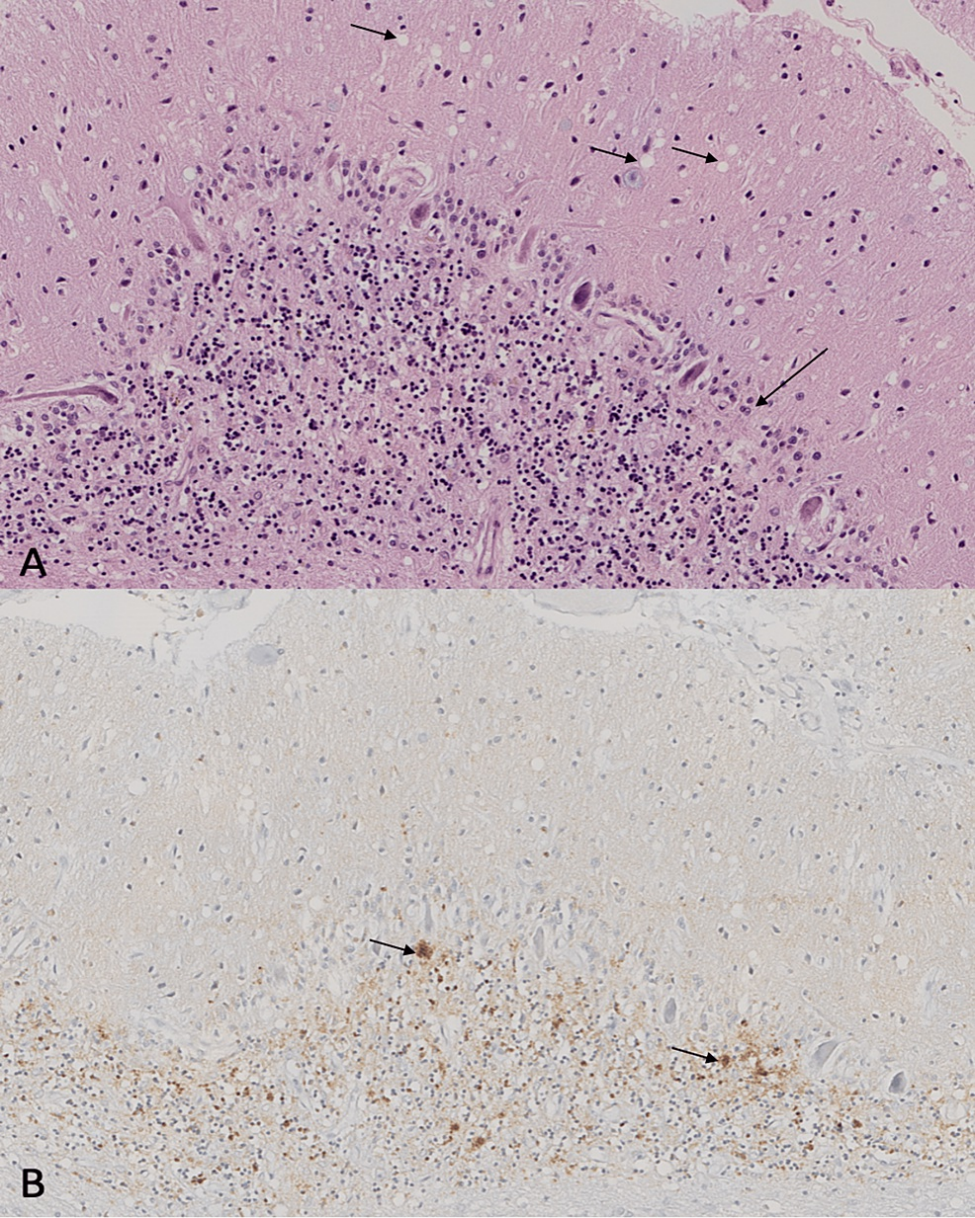
Pathology findings A: Cerebellar cortex (hematoxylin and eosin, x15), with small- and medium-sized vacuoles in the molecular layer (short arrows); loss of Purkinje cells and gliosis (long arrow). B: Cerebellar cortex (prion protein, x12), with a granular- and plaque-type (short arrows) reaction in the granular layer.

The patient’s clinical state deteriorated rapidly and she died. Family consent was obtained, and a brain biopsy was performed revealing mild generalized atrophy. Microscopically, there was a spongiform change with exuberant vacuolization in the neuropil involving layers IV-VI of the neocortex most evident in the parietal regions. Basal ganglia and thalamus had moderate vacuolization, whereas the superior colliculus had moderate to severe vacuolization. There was also mild to moderate gliosis in all cerebral regions (Figure 1A). The immunohistochemical evaluation with antibodies for prion proteins showed granular-type and plaque-type immunoreactivities in the neocortex, striatum, and cerebellum (Figure 1B). These morphological and immunophenotypic findings confirmed the diagnosis of sporadic CJD.

## Discussion

Rapidly progressive dementias are distinguished from the more typical dementias by a subacute time course and an accelerated rate of decline that develops in less than two years. The most concerning differential diagnosis in any patient presenting with this form of dementia is CJD, a fatal prion-related neurodegenerative illness [10].

CJD is a rare and untreatable disease that has been divided into various types, namely, sporadic, iatrogenic, genetic, and variant [4]. In this case, the patient had sporadic CJD, which is the most common. The mean age of onset is usually a lot lower than the age of this patient, but some cases have also been reported in older patients over 80 years of age [5].

Diagnosis can present a great challenge, since typical clinical features are non-specific and can be present in other differential neurodegenerative diseases. The diagnosis was considered probable based on the criteria from the Center for Disease Control and Prevention (CDC), supported by clinical findings and cerebrospinal fluid analysis [11]. Common clinical findings include rapid cognitive and functional decline, memory deficit, personality change, pyramidal/extrapyramidal and cerebellar signs, visual deficits, and myoclonus and akinetic mutism [6]. As described, many of these findings were present in our patient, whose cognitive and functional state declined rapidly culminating in bed confinement and mutism, with a total dependency in daily life activities.

Imaging studies, electroencephalography, and biomarkers are also very useful in the corroboration of the diagnosis [8]. This patient was submitted to a brain CT scan and MRI, but neither of these exams revealed any alterations that supported the diagnosis. In the EEG, isolated and periodic biphasic and triphasic waves with one to two second repetitions were present, which is a common finding in this disease. Finally, the CSF analysis was positive for protein 14.3.3, with elevated total tau protein and normal phosphorylated TAU protein, which, although not very sensitive, supported the diagnosis.

This patient had common clinical findings, an EEG with characteristic wave patterns and CSF that was positive for known typical biomarkers. Furthermore, routine investigations did not suggest an alternative diagnosis, making CJD a probable diagnosis.

Definitive diagnosis requires pathological confirmation by brain biopsy or autopsy [8]. Macroscopic examination of the brain usually shows no specific findings due to rapid progression from symptoms to death. The three main histological features that characterize CJD are spongiform change (vacuolization), neuronal loss, and astrogliosis. Confirmation of accumulation of abnormal prion protein is done by immunohistochemistry [3]. Because the patient died in the hospital, due to pneumonia complications, it was possible to send the brain for biopsy, where typical findings were present, confirming the diagnosis.

## Conclusions

This case of dementia in an 84-year-old woman showed clinical, electrophysiological, and cerebrospinal fluid findings suggestive of CJD, a rare, incurable, and fatal disease. Early and accurate diagnosis can represent a challenge for any clinician since there are many other entities that are more common and have similar presentations. Early diagnosis is important because it will allow patients and their families to prepare for the expected disease course and to manage expectations.

The aim of this case report is to highlight the significance of a disease that is lethal and can sometimes go unnoticed and also the importance of the differential diagnosis of dementia.
